# An On-Board Pure H_2_ Supply System Based on A Membrane Reactor for A Fuel Cell Vehicle: A Theoretical Study

**DOI:** 10.3390/membranes10070159

**Published:** 2020-07-21

**Authors:** Payam Parvasi, Seyyed Mohammad Jokar, Angelo Basile, Adolfo Iulianelli

**Affiliations:** 1 Department of Chemical, Petroleum and Gas Engineering, Shiraz University of Technology, Shiraz 71345, Iran; jokar@sutech.ac.ir; 2 Institute on Membrane Technology of the Italian National Research Council (CNR-ITM), Via Pietro Bucci, Cubo 17/C, 87036 Rende (CS), Italy; a.basile@itm.cnr.it

**Keywords:** fuel cell vehicle, hydrogen, on-board Pd–Ag membrane reformer, methane steam reforming reaction

## Abstract

In this novel conceptual fuel cell vehicle (FCV), an on-board CH_4_ steam reforming (MSR) membrane reformer (MR) is considered to generate pure H_2_ for supplying a Fuel Cell (FC) system, as an alternative to the conventional automobile engines. Two on-board tanks are forecast to store CH_4_ and water, useful for feeding both a combustion chamber (designed to provide the heat required by the system) and a multi tubes Pd-Ag MR useful to generate pure H_2_ via methane steam reforming (MSR) reaction. The pure H_2_ stream is hence supplied to the FC. The flue gas stream coming out from the combustion chamber is used to preheat the MR feed stream by two heat exchangers and one evaporator. Then, this theoretical work demonstrates by a 1-D model the feasibility of the MR based system in order to generate 5 kg/day of pure H_2_ required by the FC system for cruising a vehicle for around 500 km. The calculated CH_4_ and water consumptions were 50 and 70 kg, respectively, per 1 kg of pure H_2_. The on-board MR based FCV presents lower CO_2_ emission rates than a conventional gasoline-powered vehicle, also resulting in a more environmentally friendly solution.

## 1. Introduction

**Figure 1:**
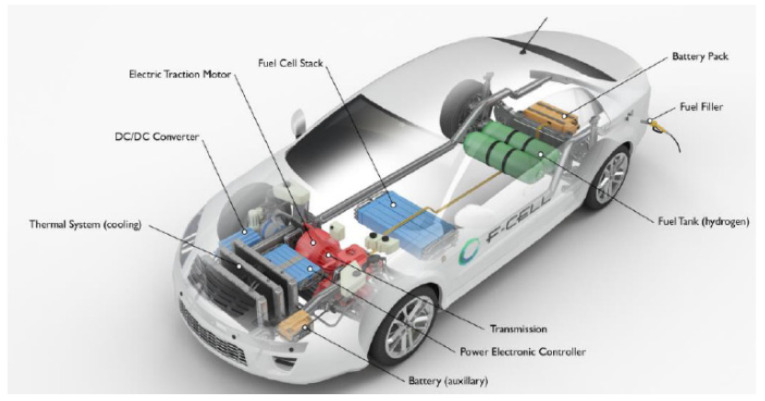
Conceptual scheme of a fuel cell vehicle (FCV) provided by a H_2_ pressurized tank [2].

Nowadays, fuel cell vehicles (FCVs) supplied by pure H_2_ are considered as a viable alternative to the internal combustion engine (ICE) powered vehicles [1]. In a H_2_ powered FCV, H_2_ reacts with O_2_ to generate power that is further transformed into mechanical energy, Figure 1.

Proton exchange membrane fuel cells (PEMFCs) are the most used fuel cells typology in transportation applications [1,2]. They are commonly supplied by pure H_2_, provided from an external source and stored in a pressurized tank [2]. With respect to the common ICE powered vehicles, a FCV presents several advantages including silent operations, lower temperature, rapid start-up, lower leakage and corrosion concerns, and lower greenhouse gases (GHGs) emission [3]. Typically, about 4–7 kg of pure H_2_ are stored in a FCV by means of pressurized tanks [1,2]. In particular, 4 kg of H_2_ enables a FCV driving-range autonomy without refueling for around 320 km [1].

Nevertheless, some open issues still remain regarding the adoption of FCVs implementing an on-board H_2_ production or a H_2_ storage system, both presenting drawbacks needing further development prior to be proposed at larger scale [4]. In this regard, the U.S. Department of Energy (DOE) first established, and it is still under development, the on-board H_2_ storage program [5], also to introduce new methods to meet the needs of the customers. Another important aspect of research is related to low energy-density of H_2_, which is responsible for its difficult storage in a car. Indeed, too large or too heavy H_2_ pressurized tanks would be required to ensure an adequate driving-range as currently guaranteed by the conventional ICEs vehicles [4]. Consequently, the on-board H_2_ generation via fuel reforming processors seems to be a quite attractive option, particularly if MSR reformers are used to generate H_2_, because this solution could exploit the CH_4_ pipeline infrastructure still existing for CH_4_ fueled ICE vehicles, even though further complexity would be added to the system and an increase of the costs as well. The former issues were analyzed in deep by Ma and Spataru [4]. Different studies in literature address the development of on-board H_2_ generation systems using different kind of fuels such as methanol, ethanol, and other hydrocarbons. Boettner and Moran [6] and Wu et al. [7] worked on the on-board FCVs development, implementing methanol fuel processors for H_2_ generation, and they demonstrated the economic advantage of this solution in terms of capital cost with respect to FCVs adopting a direct H_2_ supplying by tank. Purnima and Jayanti [8] simulated the on-board reforming of ethanol for a FC powered vehicle, also coupling a methane reformer to provide the heat of ethanol reforming reaction. They demonstrated that an overall efficiency of a bit less than 50% could be reached by the proposed system. Zhang et al. [9] analyzed the use of dimethyl ether (DME) as a fuel for on-board reformer in a PEM fuel cell system. They proved that the DME reformer could supply acceptable hydrogen for a FCV [9]. An on-board n-heptane fuel processor system was simulated for driving a 2–3 kW FCV by Karakaya and Avci [10], coupling a methane combustor also for supplying the required heat for n-heptane steam reforming. Darwish at al. [11] investigated the feasibility of hydrogen generation in a 50 kW FCV by on-board naphtha reforming process. They founded that a 70 L fuel tank should be required to guarantee 5 h of continuous driving time due to a consume of 14 L/h of naphtha in the reformer. Myers et al. [12] pointed out how MSR represents an efficient process for H_2_ generation for FCVs supplying, highlighting the following advantages: (1) N_2_ dilution effect could be ignored and, consequently, the highest H_2_ generation may be attained by MSR reaction among the other H_2_ generation methods (autothermal reforming and partial oxidation); (2) the high temperature of the output gas stream could provide the heat inputs required, (3) no need to compress air to the system because the reaction pressure could be achieved by pumping the reactants; (4) the CH_4_ price is consistently lower than that of other fuels.

To the aforementioned examples, a consistent number of research studies was dedicated in the last decade to find alternative solutions to the conventional reformers under the purpose of pursuing the principles of Process Intensification Strategy (PIS) [13]. In this regard, the role of membrane engineering in the application of PIS in the fuel reforming was largely studied, demonstrating several advantages over the conventional technologies [14,15,16,17,18,19,20,21]. In the field of H_2_ generation, many studies were dedicated to the conversion of CH_4_ into pure H_2_ via reforming reactions in membrane reformers (MRs), highlighting the operational and economic benefits over the conventional reformers [16,17,18,22,23,24,25]. In particular, metallic membranes were largely studied in MR applications, with palladium and its alloys resulting the dominant materials for preparing inorganic membranes due to the high solubility and permeability of H_2_ through them [19,20,26,27,28,29,30,31].

The H_2_ produced in a fully H_2_ perm-selective Pd-based MR is hence directly useful for a PEMFC supply without needing any additional H_2_ purification stage. This constitutes the superiority of an on-board Pd-based MR adoption over an on-board conventional reformer, which would require further H_2_ purification stage processes to purify the reformed H_2_-rich stream in order to meet the strict purity requirements of a PEMFC (CO content below 10 ppm).

The H_2_ transport through a dense palladium or palladium-alloy film occurs in six stages under a driving force (from a high to a low pressure gas region): (a) diffusion of molecular H_2_ at the Pd membrane surface, (b) reversible dissociative adsorption on the Pd surface, (c) dissolution of atomic H into the bulk metal, (d) diffusion of atomic H through the bulk metal, (e) association of H atom on the Pd surface, (f) desorption of molecular H_2_ from the surface, (g) diffusion of molecular H_2_ away from the surface [20].

Hence, the H_2_ permeation through Pd-based membranes is generally described by the Sieverts–Fick law (1):

(1) $$J_{H_{2}}=\frac{Q\left(p_{hps}^{0.5}-p_{lps}^{0.5}\right)}{\delta }$$

where, *J_H_*_2_ represents the H_2_ permeating flux, *Q* the H_2_ permeability, *δ* the thickness of the palladium/palladium alloy film, *p_hps_* and *p_lps_* are the H_2_ partial pressures on the high pressure (feed) and low pressure (permeate) sides, respectively, while “0.5” is the Sieverts pressure exponent, representing the bulk diffusion controlling step of the H_2_ permeation mechanism [21].

Several applications are noticed in literature regarding the application of Pd-based MRs to carry out MSR reaction for generating pure H_2_ to be directly supplied to PEMFCs (with standard requirements of highly H_2_ concentrated streams showing CO concentration below 10 ppm) [5,8,16,18,19], reaching high CH_4_ conversions generally at lower temperatures than the equivalent conventional reformers [17,32] due to the selective permeation of H_2_ through the membrane that is responsible for the shift of MSR reaction towards the products, enhancing both the conversion and H_2_ yield [16,22,23,24,25]. Although a large body of literature on MRs for H_2_ for generation may be noticed, to our best knowledge there are no applications of FCVs adopting MRs on-board. This choice would result more convenient also than a solid oxide electrolysis cell (SOEC) utilization for generating pure hydrogen. Indeed, the former shows as main issue (still unsolved) the high degradation under a longer operation, which would result as a limit for ensuring stable and long vehicle cruising. The SOEC degradation may occur in cell components such as hydrogen/air electrodes and electrolytes due to structural, electrochemical, and thermal modifications in the components, which in turn would affect the SOEC performance.

The novelty of this work consists of the modeling of an on-board Pd-Ag based multi-tubes MR used for generating 5 kg/day of pure H_2_ from MSR reaction to be supplied to a FCV (in this case, a pure CH_4_ feed stream was assumed without sulphur based odorants as-on the contrary-normally present in the already existing CH_4_ refueling stations). As the diffusion could be neglected in angular direction at low pressures [26,27], a 1-D model was adopted to evaluate macroscopically the size, the efficiency, and the hydrogen generation performance of the proposed on-board membrane-based fuel processor as a viable option for the FCVs development.

## 2. On-Board Processor Description

**Figure 2:**
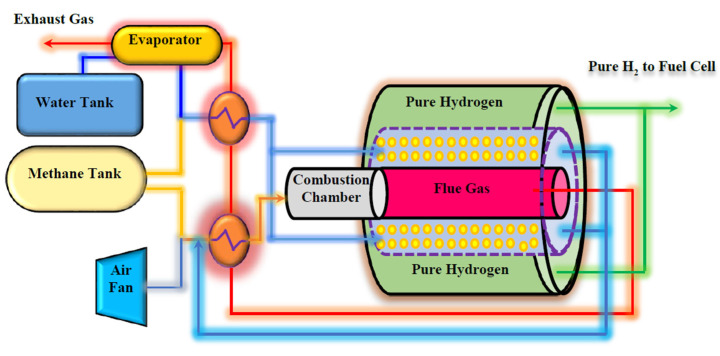
The proposed system for pure hydrogen producing in an on-board engine.

Figure 2 shows the schematic diagram of the proposed Pd-Ag MR on-board to produce pure H_2_ from MSR reaction for a FCV application.

Two separate tanks are embedded to provide water and CH_4_ for the system. MSR reaction is carried out in a multi-tubes MR packed with a Ni-based catalyst, adopting tubular unsupported Pd-Ag membranes, 50 µm thick. The heat required for carrying out the MSR reaction is supplied by burning a portion of the CH_4_ in a combustion chamber, placed before the MR. The combustion chamber produces high temperature flue gas, giving the possibility of generating steam and hot combustion air by flue gas heat exchanging with water and air in heat exchangers and evaporators. The produced steam and hot air are used in the MR.

## 3. Mathematical Modeling

This theoretical study evaluates the feasibility of the proposed on-board MR and the entire FCV system, which is modeled at steady state and non-isothermal conditions.

### 3.1. Combustion Chamber Mass and Energy Balances

**Figure 3:**
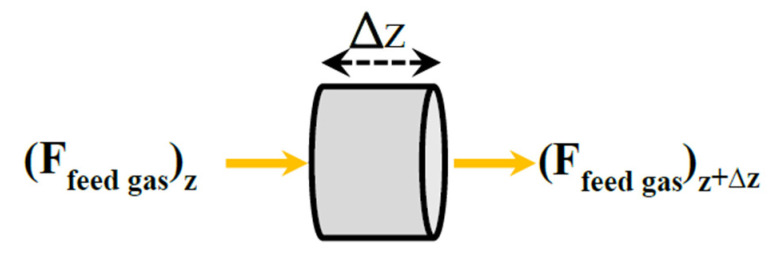
An elemental volume of the combustion chamber.

The differential volume element of thickness Δ*z* for the combustion chamber is shown in Figure 3.

The combustion process is assumed complete. The mass and energy balances are written for an adiabatic reactor.

(2) $$\frac{dF_{i}}{dz}=\sum_{j}\eta r_{j}\rho_{B}\;i=CH_{4},H_{2}O,CO_{2},\;O_{2}$$

(3) $$\frac{dT_{combustion}}{dz}=\frac{r_{combustion}(-\Delta H_{f})\eta \rho_{B}A_{C}-\sum_{i=1}^{4}Cp_{i}\frac{dF_{i}}{dz}(T_{combustion}-T_{ref})}{\sum_{i=1}^{4}Cp_{i}F_{i}}$$

where *F_i_* is the molar flow rate of component *i*, *η* is catalyst effectiveness factor, *r_j_* is rate of reaction of component *j*, *ρ_B_* is the bed density, *T_combustion_* is the combustion chamber temperature, ∆*H_f_*, is the enthalpy change of combustion reaction and *A_C_* is the cross area of the chamber. The boundary conditions of combustion chamber are as following:

(4) $$z=0\;;\;F_{i}=F_{i\;feed1},\;T_{combustion}=T_{feed1}$$

where *F_i feed_*_1_ and *T_feed_*_1_ are the molar flow rate of component *i* and the temperature of the combustion chamber feed.

### 3.2. Reformer Mass and Energy Balances

**Figure 4:**
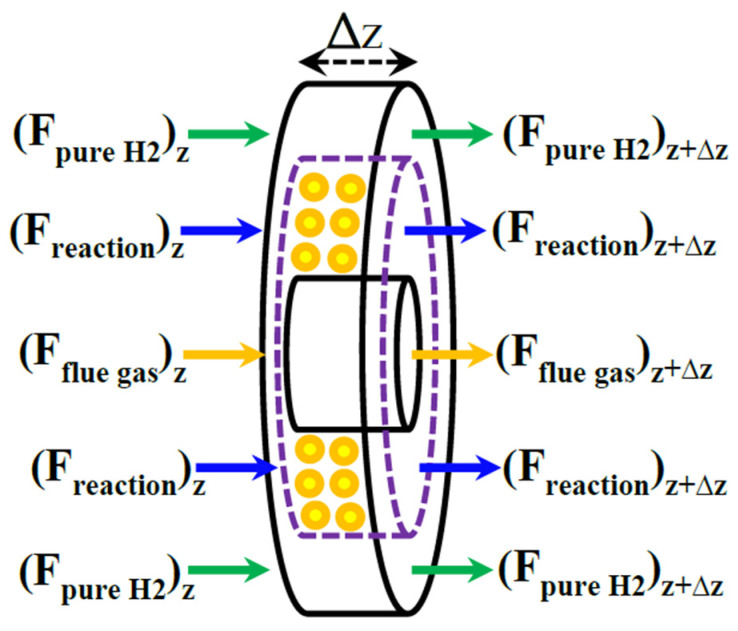
An elemental volume of the membrane reformer (MR) during MSR reaction process.

The Pd-Ag MR packed with a Ni-based catalyst is modeled on the basis of an elemental volume of the MR, as shown in Figure 4.

It may be schematized in three zones: reaction side, membrane side, and heating media (flue gas zone).

#### 3.2.1. Reaction Side

The reaction side mass balance is written according to Equation (2).

Equation (5) shows the expression to calculate the H_2_ permeation flux ($J_{H_{2}}$):

(5) $$J_{H_{2}}=\frac{Q_{Pd-Ag}}{\delta }({p_{H_{2}}^{r}}^{0.5}-{p_{H_{2}}^{m}}^{0.5})$$

In this equation, $p_{H_{2}}^{r}$ and $p_{H_{2}}^{m}$ are H_2_ partial pressures in the reaction and membrane sides, respectively. Furthermore, δ and $Q_{Pd-Ag}$ are the Pd-Ag membrane thickness and H_2_ permeability, respectively.

For the dense Pd-Ag membrane $Q_{Pd}$ is determined by [28], assuming also its full H_2_ perm-selectivity:

(6) $$Q_{Pd-Ag}=\frac{p_{0}}{\delta }exp\left(-\frac{E_{0}}{RT}\right)$$

where *p*_0_ is the pre-exponential factor (6.82 × 10^−5^ $\frac{mol}{m^{2}s\sqrt{bar}}$) and *E*_0_ is the apparent activation energy (13,412 j/mol). R is the universal gas constant, and *T* is the bulk temperature.

By considering the poisoning effect of possible byproducts formed during MSR reaction such as CO or CO_2_ on the H_2_ permeability of the dense Pd-Ag membrane, the following equations developed by Perez et al. [28] were considered:

(7) JH2CO or CO2=JH2×JH2*

(8) $${J_{H_{2}}}^{*}=\left(1-\alpha \frac{K_{i}{\bar{p}}_{i}}{1+K{\bar{p}}_{i}}\right)$$

In Equations (7) and (8), JH2CO or CO2 and ${j_{H_{2}}}^{*}$ are the H_2_ permeation flux in the presence of CO or CO_2_ and the normalized flux of H_2_, respectively. α is a dimensionless parameter depending only on the temperature (it accounts for additional effects of the adsorbed gas), $K_{i}$ the adsorption equilibrium constant (Pa^−1^), and ${\bar{p}}_{i}$ average partial pressure (Pa) of species *i*. Table 1 shows the quantities of *α* and *K_i_* at different temperatures. At T > 400 °C, the CO effect on H_2_ permeability is not comparable with the CO_2_ one and, at T > 450 °C, also the CO_2_ effect results to be negligible [28].

By considering the Equations from (5) to (8), the H_2_ flow rate in the reaction side could be calculated by the following equation:

(9) $$\frac{dF_{H_{2}}^{r}}{dz}=\underset{j=1}{\sum }\eta r_{j}\rho_{b}+\frac{Q_{Pd}\cdot 2\pi \cdot \left(r_{0}+\delta \right)}{\delta }\left(1-\alpha \frac{k_{i}{\bar{p}}_{i}}{1+k_{i}{\bar{p}}_{i}}\right)\left({p_{H_{2}}^{r}}^{0.5}-p_{H_{2}}^{m}0.5\right)$$

The energy balance of the reaction side could be written as follows:

(10) $$\frac{dT_{reac}}{dz}=\frac{\eta \rho_{B}\sum_{j=1}^{4}r_{j}(-\Delta H_{f,j})A_{C}-\sum_{i=1}^{6}Cp_{i}\frac{dF_{i}}{dz}(T-T_{ref})+\pi D_{so}U_{shell}(T_{flue\;gas}-T_{reac})}{\sum_{i=1}^{6}Cp_{i}F_{i}}$$

where *T_react_* is the reaction side temperature, ∆*H_f_*_,*j*_ is the enthalpy change of reaction *i, A_s_* is the cross area of reaction side, and *D_so_* is the external diameter of the reaction side.

The following boundary conditions should be applied:

(11) $$z=0\;;\;F_{i}=F_{i\;feed},\;T_{reac}=T_{heat-exchanger_{-}exit}$$

#### 3.2.2. Membrane Side

The membrane side mass balance is:

(12) $$\frac{dF_{H_{2}}^{m}}{dz}=\frac{Q_{Pd}\cdot 2\pi \cdot \left(r_{0}+\delta \right)}{\delta }\left(1-\alpha \frac{k_{i}{\bar{p}}_{i}}{1+k_{i}{\bar{p}}_{i}}\right)\left({p_{H_{2}}^{m}}^{0.5}-{p_{H_{2}}^{r}}^{0.5}\right)$$

The boundary conditions for the membrane side are as follows:

(13) $$z=0\;;\;F_{H_{2}}=F_{H_{2}\;sweep}$$

#### 3.2.3. Flue Gas Side

The energy balance of the flue gas could be written as follows:

(14) $$\frac{dT_{flue\;gas}}{dz}=\frac{\pi D_{so}U_{shell}(T_{reac}-T_{flue\;gas})}{{\dot{m}}_{flue\;gas}C_{P_{flue\;gas}}}$$

where *T_flue gas_* is the temperature of heating media. The following boundary condition should be applied:

(15) $$z=0\;;\;T_{flue}=T_{combustion_{-}exit}$$

The overall heat transfer coefficient is given by the following correlation:

(16) $$\frac{1}{U_{shell}}=\frac{1}{h_{i}}+\frac{A_{i}\ln(\frac{D_{o}}{D_{i}})}{2\pi LK_{w}}+\frac{A_{i}}{A_{o}}\frac{1}{h_{o}}$$

where *h_i_* and *h_o_* are the heat transfer coefficients obtained by the following correlation [33]:

(17) $$\frac{h}{C_{p}\rho \mu }{\left(\frac{C_{p}\mu }{K}\right)}^{2/3}=\frac{0.458}{\varepsilon }{\left(\frac{\rho Vd}{\mu }\right)}^{-0.407}$$

where, in the above equation, *V* is velocity of gas and the other parameters are those of bulk gas phase.

### 3.3. Reaction Kinetics

Considering the Westbrook and Dryer (WD) global one step mechanism, the following reaction could be considered for the combustion chamber [34]:

(18) $$CH_{4}+2O_{2}\;\Rightarrow \;CO_{2}+2H_{2}O\;\Delta H=-802.6\;kJ/mol$$

Equation (19) addresses the concerning reaction rate for CH_4_ combustion [34]:

(19) $$R_{combustion}=2.118726\times 10^{11}e^{\frac{-35000}{RT}}{\left[\mathrm{CH}4\right]}^{0.2\;}{\left[O2\right]}^{1.3}\;\left(\mathrm{units}\;\mathrm{in}\;\mathrm{cm},\;s,\;\mathrm{cal},\;\mathrm{and}\;\mathrm{mol}\right)$$

CH_4_ conversion takes place in the MR via the steam reforming reaction over a Ni based catalyst.

(20) $$CH_{4}+H_{2}O\;\Leftrightarrow \;CO+3H_{2}\;\Delta H=+206.2\;kJ/mol$$

(21) $$CO+H_{2}O\;\Leftrightarrow \;CO_{2}+H_{2}\;\Delta H=-41.7\;kJ/mol$$

(22) $$CH_{4}+2H_{2}O\;\Leftrightarrow \;CO_{2}+4H_{2}\;\Delta H=+164.9\;kJ/mol$$

The corresponding reaction rate equations are as follows [31,32]:

(23) $$R_{1}=\frac{\frac{k_{1}}{P_{H_{2}}^{2.5}}\left(P_{CH_{4}}P_{H_{2}O}-\frac{P_{H_{2}}^{3}P_{CO}}{K_{1}}\right)}{{\left(1\;+K_{CH_{4}}P_{CH_{4}}+K_{H_{2}}P_{H_{2}}+K_{CO}P_{CO}+K_{H_{2}O}P_{H_{2}O}\;/P_{H_{2}}\right)}^{2}}$$

(24) $$R_{2}=\frac{\frac{k_{2}}{P_{H_{2}}^{3.5}}\left(P_{CH_{4}}P_{H_{2}O}^{2}-\frac{P_{H_{2}}^{4}P_{CO_{2}}}{K_{2}}\right)}{{\left(1\;+K_{CH_{4}}P_{CH_{4}}+K_{H_{2}}P_{H_{2}}+K_{CO}P_{CO}+K_{H_{2}O}P_{H_{2}O}\;/P_{H_{2}}\right)}^{2}}$$

(25) $$R_{1}=\frac{\frac{k_{1}}{P_{H_{2}}^{2.5}}\left(P_{CH_{4}}P_{H_{2}O}-\frac{P_{H_{2}}^{3}P_{CO}}{K_{1}}\right)}{{\left(1\;+K_{CH_{4}}P_{CH_{4}}+K_{H_{2}}P_{H_{2}}+K_{CO}P_{CO}+K_{H_{2}O}P_{H_{2}O}\;/P_{H_{2}}\right)}^{2}}$$

The catalyst effectiveness factors for *R*_1_, *R*_2_, and *R*_3_ are 0.01, 0.01, and 0.3, respectively, (taken from [32]). They were considered constant due to the short length of the reaction tubes (50 cm). Thermodynamic and rate constants for Equations (23)–(25) over Ni-based catalysts are given in Table 2, where reactions rates are in mol.kg^−1^.s^−1^, and the various partial pressures in bar.

### 3.4. Heat Exchangers and Evaporator Modeling

As shown in Figure 2, two heat exchangers and an evaporator are designed for preheating the CH_4_, water, and air on their way to the membrane processor. They are placed before the combustion chamber and MR. The general energy balance equations for these two exchangers are:

(34) $${\left(F_{C}C_{P_{C}}T_{C}\right)}_{0}-{\left(F_{C}C_{P_{C}}T_{C}\right)}_{L}+Q_{HEX}=0$$

(35) $${\left(F_{C}C_{P_{C}}T_{C}\right)}_{0}-{\left(F_{C}C_{P_{C}}T_{C}\right)}_{L}+Q_{HEX}=0$$

where *C_PC_* and *C_PH_* are heat capacities of cold and hot streams in heat exchangers. *T*, *F*, and *Q_HEX_* are the temperature, flow, and heat transfer rate, respectively. The general energy balance equations for evaporators are:

(36) $${\left(F_{H}C_{P_{H}}T_{H}\right)}_{L}-{\left(F_{H}C_{P_{H}}T_{H}\right)}_{0}-Q_{evap.}=0$$

(37) $${\dot{m}}_{water}H_{liquid}-{\dot{m}}_{water}H_{vapor}+Q_{evap.}=0$$

where *Q_evap._* is the heat transfer rate in the evaporator.

## 4. Numerical Solution

**Figure 5:**
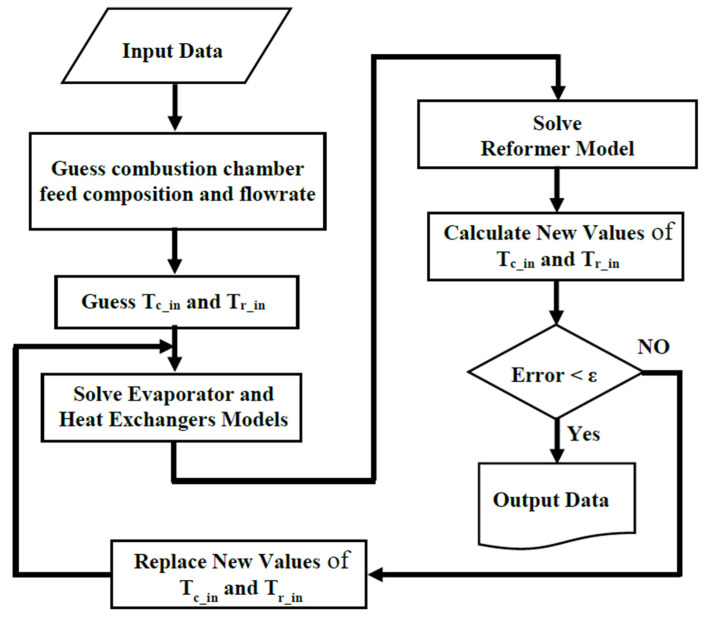
The flowchart of the program for solving the on-board MSR processor model.

Figure 5 shows the flowchart of the program developed for modeling the proposed on-board processor.

As shown, at first the initial guesses are declared. The guessing parameters are:

1. The combustion chamber inlet temperature (*T*_c,in_),
2. The reformer reaction side inlet temperature (*T*_r,in_),
3. The combustion chamber feed composition and flowrate.

The modeling codes were written by MATLAB 2016a software. The modified Rosenbrock method (ode23s) was used to solve the set of stiff ordinary differential equations (ODEs). A total of 100 nodes were considered for numerical solution of ODEs [35].

## 5. Results and Discussion

In this section, the developed model is used to investigate the loop’s performance and the impact of various parameters on it. Table 3 shows the specifications of the Toyota Mirai FCV, in which it is reported a H_2_ tank capacity of 5 kg, a H_2_ consumption in a combined cycle cruising equal to 0.76 kg_H2_/100 km, and the total cruising range for this car equal to 500 km [36].

**Figure 6:**
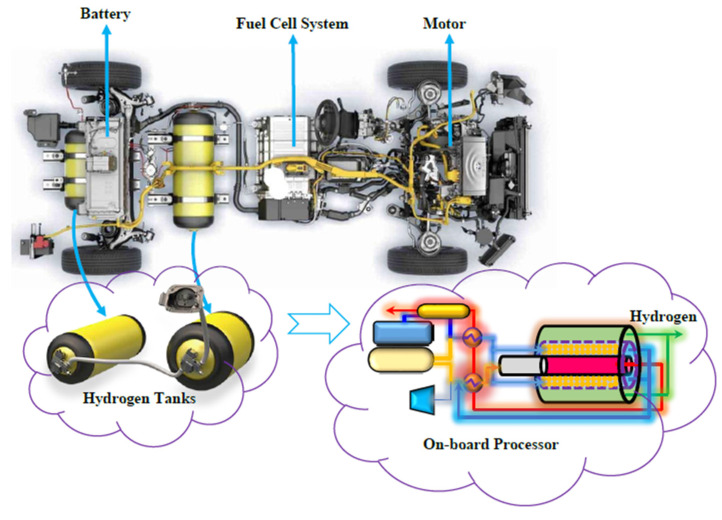
The replacement of the on-board MR combined to a H_2_ storage tank on a FCV [36].

The proposed system could be hence replaced with the proposed innovative on-board H_2_ production system (Figure 6). It should be mentioned that the methane consumption is proportional to FCV speed. Therefore, a fuel injection system should be applied to set the amount of methane injected to the on-board processor.

The operating and geometrical parameters of the on-board processor model are listed in Table 4. The parameters were chosen to give a reasonable fuel processor geometry for a five-passenger, mid-size sedan (Toyota Mirai). The membrane tubes thickness of 50 µm was chosen due to the higher permeability and lower manufacturing cost than ticker dense and unsupported membranes present in the market [30,37].

### 5.1. The Processor Performance

The methane conversion and H_2_ recovery were calculated with the following equations:

(38) $$X_{CH_{4}}=\frac{F_{CH_{4}}^{feed}-F_{CH_{4}}^{product}}{F_{CH_{4}}^{feed}}\times 100$$

(39) $$H_{2}re\mathrm{cov}ery=\frac{F_{H_{2}}^{membrane\;side}}{F_{H_{2}}^{membrane\;side}+F_{H_{2}}^{reaction\;side}}\times 100$$

The developed MR model was validated in our previous work [37]. The required feed flow rates (CH_4_, water, and air) for the system are summarized in Table 5.

**Figure 7:**
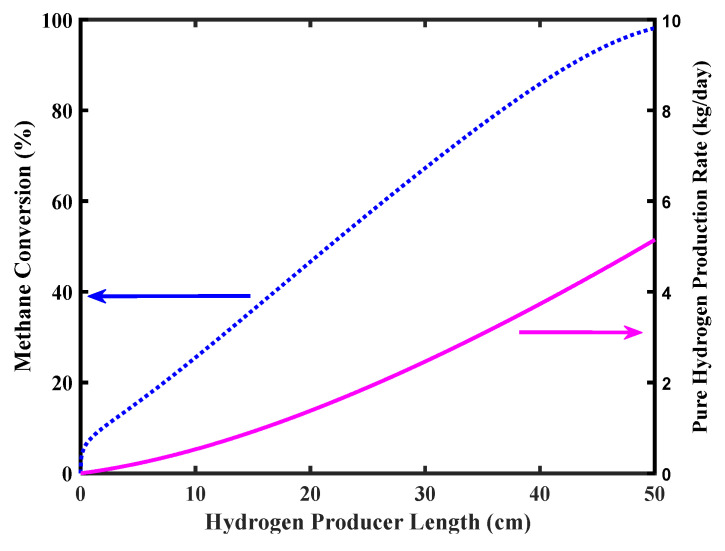
Methane conversion and pure hydrogen production rate of the MR, Pd-Ag membrane thickness = 50 µm, water to methane ratio = 2.5, inlet temperature = 500 °C, and inlet pressure = 10 bar.

Figure 7 shows CH_4_ conversion and pure H_2_ production rate versus the reformer length. Complete CH_4_ conversion is achieved in the MR outlet stream with a pure H_2_ production a bit higher than 5 kg/day as in the target of the FCV requirements.

**Figure 8:**
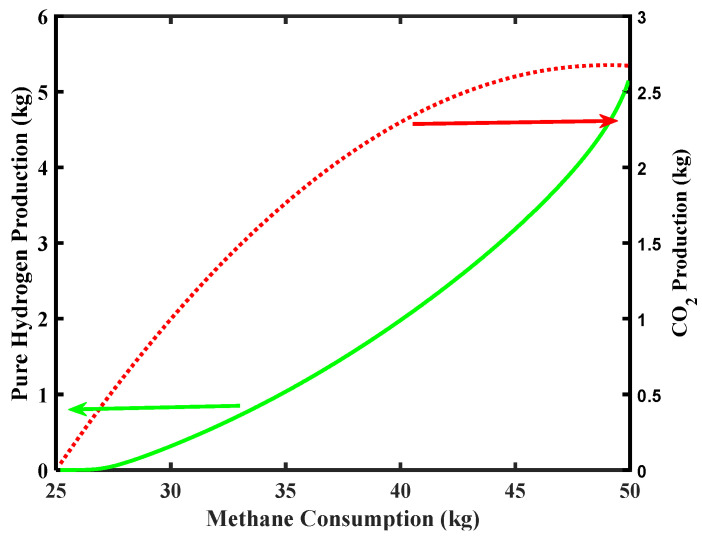
CO_2_ and pure H_2_ productions versus CH_4_ consumption for Pd-Ag membrane thickness = 50 µm and water to methane ratio = 2.5, inlet temperature = 500 °C, and inlet pressure = 10 bar.

Figure 8 reports the simulation related to CO_2_ and pure H_2_ productions along the CH_4_ consumption. Considering that the Environmental Protection Agency (EPA) report (2018) [38] sets the average CO_2_ emission for a five-passenger car equal to around 253 g/km (126.5 kg for a cruising range of 500 km), the simulation of the MR of Figure 8 shows that the maximum CO_2_ production is below this requirement in the outlet stream coming out from the MR, making the MR process theoretically feasible under the aforementioned limitations.

In addition, taking into account that the feed of CH_4_ depends on the vehicle’s H_2_ consumption, CH_4_ consumption is highly depleted for a FCV presenting lower engine power. Steam to CH_4_ mole ratio is a significant parameter affecting MSR process efficiency. This ratio ranges from 2 to 3 for industrial steam methane reformers [22].

### 5.2. The Processor Flexibility

**Figure 9:**
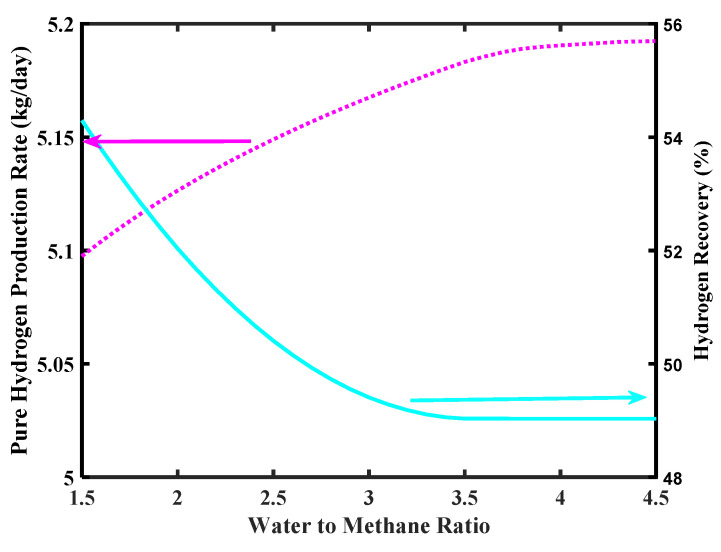
Pure H_2_ production rate and hydrogen recovery ratio versus water to methane ratio, Pd-Ag membrane thickness = 50 µm, inlet temperature = 500 °C, and inlet pressure = 10 bar.

Figure 9 shows the effect of feed ratio on pure H_2_ production rate. As shown in the simulation, the H_2_ production increases as the steam to CH_4_ mole ratio increases, even though values higher than 4 do not affect significantly the H_2_ production rate, meanwhile overcoming 5 kg/day as requested by the FCV target. On the contrary, the H_2_ recovery decreases as a consequence of a steam/CH_4_ ratio increase. Indeed, at higher steam/CH_4_ ratio the amount of steam in excess lowers the H_2_ partial pressure in the reaction side, globally determining a lower H_2_ permeation driving force, which involves a lower amount of H_2_ collected in the permeate side.

**Figure 10:**
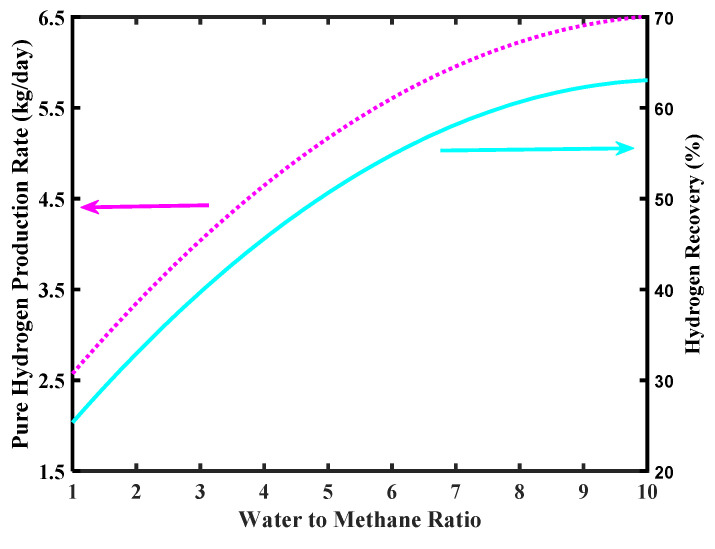
Pure H_2_ production rate and hydrogen recovery versus MR pressure, Pd-Ag membrane thickness = 50 µm, water to methane ratio = 2.5, and inlet temperature = 500 °C.

The simulations of Figure 10 show the effect of an increase of pressure on the H_2_ production rate and H_2_ recovery. In both cases, a higher pressure favors an increase of production rate and the recovery. This because a higher pressure determines a larger H_2_ permeation driving force, which enhances CH_4_ conversion due to an improved “shift effect” on the reaction system, with consequent larger H_2_ production. For the same reason, more H_2_ is collected in the permeate stream and the recovery is consequently improved. Nevertheless, unsupported Pd-based membranes suffer relatively high pressure due to structural limits. Therefore, realistically, an operating pressure with unsupported Pd-based membranes should not overcome 5–6 bar.

A technical solution for this issue was found by Basile et al. [39], who increased the permeate pressure to respect a total pressure difference across the membrane not higher than 5 bar. Nevertheless, the H_2_ permeation driving force depends on the H_2_ partial pressure difference and not on the total pressure difference; high H_2_ recovery was, then, achieved without structural problems for the dense unsupported Pd-Ag membrane.

However, fuel cell systems for vehicle applications are much more complex than stationary systems because of the need to accommodate load-following transients, identifying this requirement as a fundamental challenge of on-board fuel reforming [40]. In this regard, transient performance for the MRs used in on-board FCVs applications result to be still an open issue, particularly at the shout-down and restart phases. Larger experimental tests will be required for solving the aforementioned problems, which were out of the scopes of this work.

## 6. Conclusions

H_2_ storage in FCVs represents one of the most challenging issues in this field. This theoretical work studied the feasibility of implementing a novel on-board MR able to produce pure H_2_ on demand from MSR reaction. CH_4_ and water were preheated by waste flue gas and reacted within a synthesis loop equipped with a Pd-Ag MR. The results showed that the system was able to produce 5 kg/day of pure H_2_, which is suitable for a FCV cruising range of 500 km. A total of 50 kg of CH_4_ and 70 kg of water were needed as feeding reactants to generate 1 kg of pure H_2_.

The effects of various parameters such as CH_4_ consumption rate, water to methane ratio, and operating pressure on the processor performance were investigated. The model showed that large pure H_2_ production rates were reached by the Pd-Ag MR at both higher steam to CH_4_ ratio and operating pressure.

## Figures and Tables

**Table 1:** The *α* and *K*_i_ parameters of Equation (9) taken from Perez et al. [28].

*T* (°C)	CO		CO_2_	
	*α*	*K_i_*	*α*	*K_i_*
450	0.12	1 × 10^−4^	0.62	4.93 × 10^−6^
400	0.20	1.03 × 10^−4^	0.77	6.30 × 10^−6^
300	0.77	1.34 × 10^−4^	0.80	1.17 × 10^−5^
250	0.78	3.35 × 10^−4^	0.90	1.50 × 10^−5^

**Table 2:** Thermodynamic and rate constants for Equations (23)–(25) are taken from Xu and Froment [32].

$k_{1}=1.8\times 10^{-6}\exp\left(\frac{240,100}{R}\left(\frac{1}{648}-\frac{1}{T}\right)\right)\;\left(\frac{\mathrm{kmol}{.\mathrm{bar}}^{0.5}}{\mathrm{kgcat}.h}\right)$	(26)
$k_{2}=2.2{\times 10}^{-5}\exp\left(\frac{243,900}{R}\left(\frac{1}{648}-\frac{1}{T}\right)\right)\;\left(\frac{\mathrm{kmol}{.\mathrm{bar}}^{0.5}}{\mathrm{kgcat}.h}\right)$	(27)
$k_{3}=7.6\exp\left(\frac{67,100}{R}\left(\frac{1}{648}-\frac{1}{T}\right)\right)\;\left(\frac{\mathrm{kmol}{.\mathrm{bar}}^{-1}}{\mathrm{kgcat}.h}\right)$	(28)
$K_{i}=\exp\left(-\frac{\Delta G_{rxn_{i}}^{\circ }}{RT}\right)\;i=1,2\;and\;3$	(29)
$K_{CH_{4}}=1.8\times 10^{-1}\exp\left(\frac{-38,300}{R}\left(\frac{1}{823}-\frac{1}{T}\right)\right)$	(30)
$K_{H_{2}}=2.9\times 10^{-2}\exp\left(\frac{-82,900}{R}\left(\frac{1}{648}-\frac{1}{T}\right)\right)$	(31)
$K_{CO}=40.9\exp\left(\frac{-70,700}{R}\left(\frac{1}{648}-\frac{1}{T}\right)\right)$	(32)
$K_{H_{2}O}=0.4\exp\left(\frac{88,700}{R}\left(\frac{1}{823}-\frac{1}{T}\right)\right)$	(33)

**Table 3:** The specifications for Toyota Mirai FCV [36].

Parameter	Value
Maximum output	155 hp
H_2_ consumption (combined cycle)	0.76 kg _H2_/100 km
Cruising range	500 km
H_2_ tank capacity	5 kg

**Table 4:** The operating and geometric parameters of the on-board processor.

Parameter	Value
Tube length in pure hydrogen producer (cm)	50
Tube length in combustion chamber (cm)	10
Cross area of the tubes (cm^2^)	5.4
Number of tubes	25
Pressure of methane and steam in pure H_2_ producer inlet (bar)	10
Temperature of methane in pure H_2_ producer inlet (°C)	500
Pressure of methane and steam in combustion chamber inlet (bar)	1
Temperature of methane in combustion chamber inlet (°C)	25
Outer reaction side diameter in pure H_2_ producer	1”
Outer shell side diameter in pure H_2_ producer (cm)	3
Catalytic bed density (kg/m^3^)	780
Outer tube side diameter in combustion chamber (cm)	0.5
Membrane thickness (µm)	50

**Table 5:** Feed flow rates for the proposed on-board MR.

Material	Consumption Value (kg/kg pure H_2_)
Methane	50
Water	70
Air	435

## Nomenclature

*A_c_*	Cross area of reaction side (m)
*C_pi_*	Specific heat of component *i* (J kmol^−1^K^−1^)
*D_So_*	External diameter of reaction side (m)
*E_0_*	Activation energy of membrane
*F_i_*	Molar flow rate of component *i*
$\Delta G_{rxn_{i}}^{\circ }$	Standard Gibbs free energy change of reaction *i*
∆*H_f,i_*	Enthalpy change of reaction *i*
*k_i_*	Reaction rate constant
*K_i_*	Adsorption equilibrium constant
*P_i_*	Partial pressure of component *i* (bar)
*r*_0_	Membrane tube radius (m)
Q_Pd_	Hydrogen permeability
*r_j_*	Rate of reaction of component *j* (kmol m^−3^ s^−1^)
*R*	Universal gas constant (J kmol^−1^ K^−1^)
*T_combustion_*	Reaction side temperature (K)
*T_react_*	Reaction side temperature (K)
*T_shell_*	Temperature of heating stream (K)
*U_shell_*	Overall heat transfer coefficient (W m^−1^ s^−1^)
$X_{CH_{4}}$	Methane conversion
$X_{CO_{2}}$	Carbon dioxide conversion
*z*	Axial direction
δ	Membrane thickness
η	Catalyst effectiveness factor
$\rho_{B}$	Bed density (kg m^−3^)
*DME*	Dimethyl ether
*DOE*	Department of energy
*FCV*	Fuel cell vehicle
*GHG*	Greenhouse gases
*ICE*	Internal combustion engine
*MR*	Membrane reformer
*MSR*	Methane steam reforming
*ODE*	Ordinary differential equations
*PEMFC*	Proton exchange membrane fuel cell
*PIS*	Process intensification strategy

## References

[1] Wilberforce T., El-Hassan Z., Khatib F.N., Makky A.A., Baroutaji A., Carton J.G., Olabi A.G. (2017). Developments of electric cars and fuel cell hydrogen electric cars. Int. J. Hydrog. Energ..

[2] Manoharan Y., Hosseini S.E., Butler B., Alzhahrani H., Senior B.T.F., Ashuri T., Krohn J. (2019). Hydrogen Fuel Cell Vehicles; Current Status and Future Prospect. Appl. Sci..

[3] Achour H., Carton J.G., Olabi A.G. (2011). Estimating vehicle emissions from road transport, case study: Dublin City. Appl. Energy.

[4] Ma L., Spataru C. (2015). The use of natural gas pipeline network with different energy carriers. Ener. Strategy Rev..

[5] Qi A., Peppley B., Karan K. (2007). Integrated fuel processors for fuel cell application: A review. Fuel Process. Technol..

[6] Boettner D.D., Moran M.J. (2004). Proton exchange membrane (PEM) fuel cell-powered vehicle performance using direct-hydrogen fueling and on-board methanol reforming. J. Energy.

[7] Wu W., Chuang B.N., Hwang J.J., Lin G.K., Yang S.B. (2019). Techno-economic evaluation of a hybrid fuel cell vehicle with on-board MeOH-to-H_2_ processor. Appl. Energy.

[8] Purnima P., Jayanti S. (2016). A high-efficiency, auto-thermal system for onboard hydrogen production for low temperature PEM fuel cells using dual reforming of ethanol. Int. J. Hydrog. Energ..

[9] Zhang T., Ou K., Jung S., Choi B., Kim Y.B. (2018). Dynamic analysis of a PEM fuel cell hybrid system with an on-board dimethyl ether (DME) steam reformer (SR). Int. J. Hydrog. Energ..

[10] Karakaya M., Avci A.K. (2010). Comparison of compact reformer configurations for on-board fuel processing. Int. J. Hydrog. Energ..

[11] Darwish N.A., Hilal N., Versteeg G., Heesink B. (2004). Feasibility of the direct generation of hydrogen for fuel-cell-powered vehicles by on-board steam reforming of naphtha. Fuel.

[12] Myers D.B., Ariff G.D., James B.D. Cost and Performance Comparison of Stationary Hydrogen Fueling Appliances. Task 2 Report 2002. https://www.nrel.gov/docs/fy02osti/32405b2.pdf.

[13] Boodhoo K., Harvey A. (2013). Process intensification for green chemistry. Chem. Listy..

[14] Drioli E., Barbieri G., Brunetti A. (2017). Membrane Engineering for the Treatment of Gases: Vol. 2, Gas-Separation Issues Combined with Membrane Reactors.

[15] Bagnato G., Iulianelli A., Sanna A., Basile A. (2017). Glycerol production and transformation: A critical review with particular emphasis on glycerol reforming reaction for producing hydrogen in conventional and membrane reactors. Membranes.

[16] Iulianelli A., Liguori S., Wilcox J., Basile A. (2016). Advances on methane steam reforming to produce hydrogen through membrane reactors technology: A review. Catal. Rev. Sci. Eng..

[17] Wang H., Liu M., Kong H., Hao Y. (2019). Thermodynamic analysis on mid/low temperature solar methane steam reforming with hydrogen permeation membrane reactors. Appl. Therm. Eng..

[18] Yan Y.-F., Zhang L., Li L.-X., Tang Q. (2011). Progress in catalytic membrane reactors for high purity hydrogen production. J. Inorg. Mat..

[19] Al-Mufachi N.A., Rees N.V., Steinberger-Wilkens R. (2015). Hydrogen selective membranes: A review of palladium-based dense metal membranes. Ren. Sust. En. Rev..

[20] Yun S., Oyama S.T. (2011). Correlations in palladium membranes for hydrogen separation: A review. J. Membr. Sci..

[21] Caravella A., Scura F., Barbieri G., Drioli E. (2010). Sieverts law empirical exponent for Pd-based membranes: Critical analysis in pure H_2_ permeation. J. Phys. Chem. B.

[22] Iaquaniello G., Giacobbe F., Morico B., Cosenza S., Farace A. (2008). Membrane reforming in converting natural gas to hydrogen: Production costs, Part II. Int. J. Hydrog. Energ..

[23] Lu N., Xie D. (2016). Novel membrane reactor concepts for hydrogen production from hydrocarbons: A review. Int. J. Chem. Reactor Eng..

[24] Yücel O., Alaittin H.M. (2016). Comprehensive study of steam reforming of methane in membrane reactors. J. Energy. Resour. Technol..

[25] Jørgensen S.L., Nielsen P.E.H., Lehrmann P. (1995). Steam reforming of methane in a membrane reactor. Catal. Today.

[26] Oyama S.T., Hacarlioglu P. (2009). The boundary between simple and complex descriptions of membrane reactors: The transition between 1-D and 2-D analysis. Int. J. Hydrog. Energ..

[27] Rahimpour M.R., Samimia F., Babapoor A., Tohidian T., Mohebi S. (2017). Palladium membranes applications in reaction systems for hydrogen separation and purification: A review. Chem. Eng. Proc. Proc. Intens..

[28] Perez P., Cornaglia C.A., Mendes A., Madeira L.M., Tosti S. (2015). Surface effects and CO/CO_2_ influence in the H_2_ permeation through a Pd-Ag membrane: A comprehensive model. Int. J. Hydrog. Energ..

[29] Hamilton H. (2012). Palladium-based membranes for hydrogen separation. Platin. Met. Rev..

[30] Plazaola A.A., Pacheco T.A.D., Van S.A.M., Gallucci F. (2017). Recent advances in Pd-based membranes for membrane reactors. Membranes.

[31] Iulianelli A., Liguori S., Vita A., Italiano C., Fabiano C., Huang Y., Basile A. (2016). The oncoming energy vector: Hydrogen produced in Pd-composite membrane reactor via bioethanol reforming over Ni/CeO_2_ catalyst. Catal. Today.

[32] Xu J., Froment G.F. (1989). Methane steam reforming, methanation and water-gas shift: I. Intrinsic kinetics. Amer. Inst. Chem. Eng. J..

[33] Smith J.M. (1980). Chemical Engineering Kinetics.

[34] Acampora L., Marra F.S. (2017). Investigation by thermodynamic properties of methane combustion mechanisms under harmonic oscillations in perfectly stirred reactor. Chem. Eng. Trans..

[35] Shampine L.F., Reichelt M.W. (1977). The Matlab ODE suite. SIAM. J. Sci. Comput..

[36] Toyota Mirai Hydrogen Fuel Cell Electric Vehicle. https://h2.live/en/wasserstoffautos/toyota-mirai.

[37] Jokar S.M., Parvasi P., Basile A. (2018). The evaluation of methane mixed reforming reaction in an industrial membrane reformer for hydrogen production. Int. J. Hydrog. Energ..

[38] (2018). Greenhouse Gas Emissions from a Typical Passenger Vehicle, U.S. Environmental Protection Agency (EPA) Report. https://nepis.epa.gov/Exe/ZyPDF.cgi?Dockey=P100U8YT.pdf.

[39] Basile A., Campanari S., Manzolini G., Iulianelli A., Longo T., Liguori S., De F.M., Piemonte V. (2011). Methane steam reforming in a Pd-Ag membrane reformer: An experimental study on reaction pressure influence at middle temperature. Int. J. Hydrog. Energ..

[40] Brown L.F. (2001). A comparative study of fuels for on-board hydrogen production for fuel-cell-powered automobiles. Int. J. Hydrog. Energ..

